# Capitoline Dolphins: Residency Patterns and Abundance Estimate of *Tursiops truncatus* at the Tiber River Estuary (Mediterranean Sea)

**DOI:** 10.3390/biology10040275

**Published:** 2021-03-28

**Authors:** Daniela Silvia Pace, Chiara Di Marco, Giancarlo Giacomini, Sara Ferri, Margherita Silvestri, Elena Papale, Edoardo Casoli, Daniele Ventura, Marco Mingione, Pierfrancesco Alaimo Di Loro, Giovanna Jona Lasinio, Giandomenico Ardizzone

**Affiliations:** 1 Department of Environmental Biology, Sapienza University of Rome, 00185 Rome, Italy; dimarco.1659968@studenti.uniroma1.it (C.D.M.); giancgiacomini@gmail.com (G.G.); ferri.1533868@studenti.uniroma1.it (S.F.); margherita.silvestri22@gmail.com (M.S.); edoardo.casoli@uniroma1.it (E.C.); daniele.ventura@uniroma1.it (D.V.); giandomenico.ardizzone@uniroma1.it (G.A.); 2 CNR-IAS, Campobello di Mazara, 91021 Trapani, Italy; elena.papale@ias.cnr.it; 3 Department of Life Sciences and Systems Biology, University of Torino, 10123 Torino, Italy; 4 Department of Statistical Sciences, Sapienza University of Rome, 00185 Rome, Italy; marco.mingione@uniroma1.it (M.M.); pierfrancesco.alaimodiloro@uniroma1.it (P.A.D.L.); giovanna.jonalasinio@uniroma1.it (G.J.L.)

**Keywords:** abundance, site fidelity, Tiber River, bottlenose dolphin, *Tursiops truncatus*, capture–recapture, Mediterranean Sea

## Abstract

**Simple Summary:**

Photo-identification is a non-invasive method of identifying individual animals from natural markings present on one or more parts of the body. The dorsal fin of a dolphin is the portion that contains individually distinctive marks and the most commonly visible when an animal surfaces to breathe. The camera captures an image of the dorsal fin whenever it is sighted and based on the number of times an image is captured, a statistical method called capture–recapture is used to estimate the site fidelity and the size of the population in a defined area. Here, we applied these methods to study the bottlenose dolphins (*Tursiops truncatus*) between 2017 and 2020 at the Tiber estuary (Mediterranean Sea, Rome, Italy), a polluted major river supplying organic material to the Capitoline (Roman) coastal area and nearby regions. We identified 347 unique individuals, with different patterns of residency (i.e., the tendency of dolphins to remain in, or return to, the study area): 42 were labeled resident, 73 part-time, and 232 transient. Estimating a total population size of 529 individuals and highlighting the presence of animals with a strong site-fidelity, this study suggests developing conservation plans for this vulnerable top-predator species not only focused on areas close to the Tiber River mouths but also extended to cover a broader scale of area.

**Abstract:**

Periodic assessments of population status and trends to detect natural influences and human effects on coastal dolphin are often limited by lack of baseline information. Here, we investigated for the first time the site-fidelity patterns and estimated the population size of bottlenose dolphins (*Tursiops truncatus*) at the Tiber River estuary (central Mediterranean, Tyrrhenian Sea, Rome, Italy) between 2017 and 2020. We used photo-identification data and site-fidelity metrics to study the tendency of dolphins to remain in, or return to, the study area, and capture–recapture models to estimate the population abundance. In all, 347 unique individuals were identified. The hierarchical cluster analysis highlighted 3 clusters, labeled resident (individuals encountered at least five times, in three different months, over three distinct years; *n* = 42), part-time (individuals encountered at least on two occasions in a month, in at least two different years; *n* = 73), and transient (individuals encountered on more than one occasion, in more than 1 month, none of them in more than 1 year; *n* = 232), each characterized by site-fidelity metrics. Open POPAN modeling estimated a population size of 529 individuals (95% CI: 456–614), showing that the Capitoline (Roman) coastal area and nearby regions surrounding the Tiber River estuary represent an important, suitable habitat for bottlenose dolphins, despite their proximity to one of the major urban centers in the world (the city of Rome). Given the high number of individuals in the area and the presence of resident individuals with strong site fidelity, we suggest that conservation plans should not be focused only close to the Tiber River mouths but extended to cover a broader scale of area.

## 1. Introduction

Estimating the abundance of populations, and their spatiotemporal variability, is a key factor for ensuring biodiversity preservation and the maintenance of the integrity of the community structure and ecosystem processes [1]. In the marine environment, there are several cases of how biodiversity, particularly the loss of populations of top-predator species, may have effects on the food web and the ecosystems’ structure [2]. Many population studies are consequently intended to estimate the abundance of a particular species, to understand the baseline conditions, assess its conservation status both at local and global scales, and evaluate population-level impacts associated with habitat modifications and natural/anthropogenic pressures [3]. These assessments may then assist conservation managers in the development of preservation actions targeted at the specific threats the population is facing [4,5,6,7,8] and in the evaluation, in terms of population size, of the efficacy of such measures [9].

For the majority of delphinids, abundance estimates are particularly challenging. They are a highly mobile and often wide-ranging top-predator species, with often open-population structures, thus requiring robust analytical methods to prevent the potential of incorrect estimates associated with irregular sampling effort, variable probability of animal detection, and non-random movement patterns [10,11]. Individuals may persistently or unevenly occur in a given area, being regularly or only present for short periods of time, showing different degrees of site fidelity (i.e., tendency to return to a previously occupied place; [12]). In studies on delphinids, many site-fidelity metrics (e.g., occurrence, permanence, and periodicity; [13]) and quantification methods (e.g., proportions, category of fidelity, models, and composite indices; [13,14,15,16]) are commonly used with the intention of considering non-resident and resident individuals [17], as failures to account for the non-residents can biased abundance estimates or erroneous understandings of local ecological dynamics [18].

Bottlenose dolphins (*Tursiops truncatus*) are key components of the inshore and coastal marine biodiversity worldwide, showing an irregular distribution dependent on the characteristics of the habitat, prey availability, and anthropogenic disturbance [19,20,21]. In the Mediterranean Sea, the bottlenose dolphin is frequently spotted in areas close to the 100 m isobath [22] but can be found up till 400 m [23,24]. The overall population size is unknown; it may be in the low 10,000 s [25], but no total abundance estimates have been determined so far, as baseline data are still deficient over extended portions of the basin. The species is included in Annex IV of the priority species of the EU Habitats Directive (92/43/EEC), and in 2012, the International Union for Conservation of Nature (IUCN) classified the Mediterranean bottlenose dolphins as “Vulnerable” in the Red List, according to the criteria A2cde and based on a suspected population decline of at least 30% over the last 60 years [25]. As a mainly coastal species, the bottlenose dolphin is extremely exposed to anthropogenic pressures due to increasing human interaction within its habitats [10], and the decline in the Mediterranean is presumably related to the cumulative impact of several threats, including reduced availability of key prey due to overfishing and incidental mortality in fishing gear, human disturbances (recreational activities and shipping), pollution (noise and environmental contaminants), and marine debris [25].

Periodic assessments of the population status and trend evaluations to detect natural influences and human effects on coastal dolphins are often limited by lack of baseline information [10]. Here, we investigated for the first time the site-fidelity patterns and estimated the population size of bottlenose dolphins between 2017 and 2020 at the Tiber River estuary (central Mediterranean Sea, Rome, Italy), an ecologically important region heavily exposed to increasing disturbance by, and impact of, human activities. The presence of bottlenose dolphin in the area has been reported since 2008 (D.S.P. and G.G, personal observations), and some evidence of the distribution of groups frequenting the region is available [26]. However, no detailed published information has been produced so far, highlighting the need of better understanding the size of the population and the site-fidelity patterns of the individuals, crucial to the implementation of adequate protection measures.

In this study, we considered the tendency of dolphins to remain in, or return to, and reuse the study area and examined the abundance of the local population to offer relevant basic data that may aid future conservation plans and management actions.

## 2. Materials and Methods

### 2.1. Study Area

**Figure 1:**
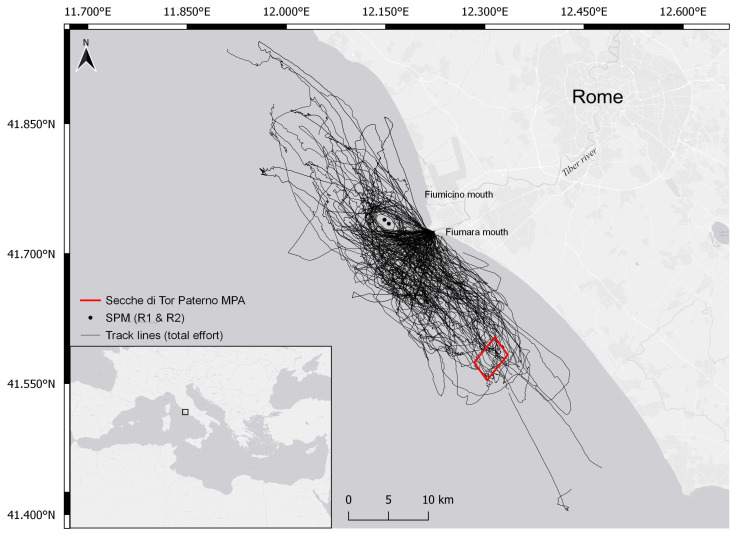
Study area in Central Tyrrhenian Sea: black lines represent the tracks made in the period 2017–2020, black dots represent single-points mooring (SPM), and the red square identifies Secche di Tor Paterno (marine protected area, MPA).

The study site (Figure 1) is located in central Mediterranean (Tyrrhenian Sea, Rome, Italy) and covers an area of approximately 1300 km^2^. It is characterized by a variety of environmental conditions and habitats, including the estuary of the Tiber River, which flows into the Tyrrhenian Sea through the two mouths, of Fiumara Grande (natural mouth) and of Fiumicino (artificial channel). The Tiber River is the main river in central Italy and the major supplier of organic material to the Capitoline (Roman) coastal area and nearby regions, contributing to the development of a rich marine community. However, crossing the city of Rome, it collects waste, organic material, and heavy metals, thus increasing the level of pollution when flowing into the sea [27,28].

The simultaneous presence of both fresh and salt waters, as well as the geomorphological action of sedimentation and erosion, generate different ecological gradients, making the area highly productive and rich in coastal biodiversity [29,30,31,32]. Portions of the seabed of the Roman coast, north and south of the two mouths of the Tiber, include habitats of biological importance (i.e., coralligenous reefs and *Posidonia oceanica* meadows), which are included in the EU Natura 2000 network Sites of Community Importance (SCI). One of these sites, Secche di Tor Paterno, IT6000010, is also a marine protected area (MPA) of 1387 ha, protected since 2000. It is the only fully submerged Italian MPA and consists of a large rocky formation that extends from 18 m up to a maximum of 60 m depth. Human activities that may be dangerous or may represent a disturbance for marine species in the MPA are forbidden.

On the banks of the Tiber close to the mouth, there are several shipyards, in addition to two touristic and fishing ports (Ostia and Fiumicino) that generate intense vessel traffic. In addition, 3 nautical miles from Fiumara Grande, there are two single-point moorings (SPMs) for the reception of crude oil and supply petroleum products (called R1 and R2); navigation, anchoring, diving, and fishing activities at a distance of 750 m from each are prohibited. Such types of structures are known to be suitable habitats and aggregation points for demersal fish species and may be exploited by bottlenose dolphins as feeding sites [26,33]. In the study area, the regular presence of groups of bottlenose dolphins is reported [26], possibly facilitated by the ecological conditions and the heterogeneity of morphological features. The region appears a suitable site for foraging and nursing bottlenose dolphins [26] and it is a valuable ground for commercial fishery (small-scale artisanal fishery and larger trawling vessels; [30]). The main targets of fishing are demersal species, and these may be the main prey for bottlenose dolphins in the Mediterranean [34]. Indeed, interactions among fishery and bottlenose dolphins are commonly observed in the area [26], as in other Mediterranean regions [35,36], but no data are available to quantify the effects in the study area, both on the commercial activity and on the dolphin population.

### 2.2. Data Collection

Boat-based daily surveys were conducted onboard a sailing vessel *Beneteau Oceanis 41.1* powered by a 55 hp Volvo diesel engine, in favorable weather conditions (i.e., sea state ≤3 Douglas, wind force ≤3 Beaufort, no rain, no fog) to reduce the detection probability bias. More than 90% of the surveys occurred in sea state ≤2 and wave height ≤0.3 m. The sampling effort was conducted in 4- to 6-month sampling periods over 4 years (2017–2020), principally in spring, summer, and early autumn. Surveys were conducted from 08:30 to 16:30 h (depending on suitable conditions) by three to six observers alternating between 7 × 50 binoculars and the naked eye, at a steady speed of 4–6 knots. Survey track lines did not follow a standardized layout (i.e., sawtooth or grid pattern lines) but a random sampling procedure [20,21] with respect to the coast and depth contours; the presence of fishing vessels/gears operating or placed in the area was also considered in order to maximize encounter rates.

A group of dolphins was defined as two or more individuals with a relatively close spatial cohesion (i.e., each member within 100 m of any other member) involved in similar (or the same) behavioral activities [4]. Upon sighting a group, the survey effort was suspended and the vessel departed from its route to approach the dolphins in order to (a) identify the species’ predominant behavior (i.e., behavioral state in which more than 50% of the animals are involved; [37]), group size, and group composition (i.e., adults, juveniles, and calves/immatures; see below for definitions) and (b) collect photographs of individual animals for identification purposes. Two observers collected photographs of the dorsal fins of all dolphins in the sighted group using Canon digital 5D and 6D cameras and Canon 100–400 mm f/4.5–5.6 L lens. Dolphins were photographed regardless of their level of marking. Once observers were confident that the best possible good-quality photographs had been obtained, or the animals were lost, the dolphin sightings ended.

At the location of each group, GPS (global positioning system) coordinates, time, direction, and the presence of concomitant anthropogenic activities (fishing vessels, fishing gears, pleasure boats, etc.) were recorded. Total group size and age class composition were estimated in the field, then corrected (if needed) via photo-identification analysis. Age class was defined as follows: adult = an individual generally of a length of about 2.8–3.0 m; juvenile = a poorly scarred and rarely nicked individual of about ⅔ the length of an adult; calf = an individual of about ½ the length of an adult, with often visible fetal folds, always swimming close to an adult in a position just behind the dorsal fin; and newborn = an individual of about ⅓ the length of an adult, with visible fetal folds, swimming uncoordinatedly always very close to an adult. Sex was determined whenever possible through photographs of the genital area or observations of constant adult–offspring associations during one or more encounters (the adult was assumed to be a female).

### 2.3. Data Analysis

#### 2.3.1. Photo-Identification

When using photographic identification as the capture–mark–recapture method for abundance estimates, two possible main sources of bias have to be addressed: the heterogeneity in capture probability and individual misidentification [38,39]. The first issue may be controlled through grading of photographs for quality, the second via scoring of individuals for distinctiveness. Here, photographs were processed considering their quality (level of sharpness/brightness; focus, angle, and visibility of the dorsal fin; perpendicularity to the axis of the dolphin’s body [40]). A quality rating (Q) between 1 (low quality) and 5 (high quality) was assigned to each image and only photos with a Q ≥ 4 were used for the analysis.

The occurrence and position of permanent natural markings (such as nicks and notches) on the leading and trailing edges of the dorsal fins (visible from both sides) were used to uniquely recognize individual dolphins [41,42]. The individual distinctiveness was scored as (a) well marked (WM): highly distinctive fin (missing tops, large nicks/notches); (b) fairly marked (FM): moderately distinctive fin (multiple small notches/nicks); and (c) unmarked (UM): indistinctive fin (no distinctive features on dorsal fin). Three different observers independently scored each individual according to the above criteria. Calves were not considered as they often lack distinctive markings and due to their dependence on their mothers, their captures are not independent, violating common model assumptions that captures are independent [43]. A selection of the best photographs of each individual was retained to set up the photo-identification catalog. To develop capture histories of individuals and for all subsequent analyses, we used only images of distinctive adult individuals deemed to be of excellent and good quality [44].

#### 2.3.2. Site Fidelity

A hierarchical cluster analysis was used to describe the presence of possible dolphin groups according to their site-fidelity patterns in the surveyed area, with the aim of identifying resident, transient, and (eventually) part-time individuals considering their encounter histories. Clusters were characterized using different composite indices [13,18] and their centroids. From a geometrical perspective, cluster centroids provide a general measure of the cluster location in the space of the considered metrics. From a practical perspective, each centroid can be seen as the cluster average individual (not necessarily a member of the data set), i.e., the theoretical unit that best represents the cluster’s typical features. Therefore, it can be used to characterize the cluster and interpret the corresponding results.

To calculate the indices, we first defined *t*_1_, . . . , *t_nocc_* as the times of the *n_occ_* capture occasions; we also labeled as *M_k_*, *k* = 1, . . . , *n_m_* and *Y_k_*, *k* = 1, . . . , *n_y_* the sets of capture occasions belonging to the *k*-th month or year, respectively. We further defined *n_d_* as the number of identified dolphins and $\;{\left\{c_{ij}\right\}}_{j=1}^{n_{occ}}$ as the encounter history associated with dolphin *i* = 1, . . . , *n_d_*, where *c_ij_* = 1 if dolphin *i* was captured on occasion *j* and 0 otherwise. Hence, the quantity $c_{i}=\sum_{j=1}^{n_{occ}}c_{ij}$ represents the total number of times the *i*-th identified dolphin was captured. Then, we calculated the times of the first and last captures of each dolphin as

$$t_{i}^{f}=\min\left\{t_{j},j=1,\ldots ,n_{occ}:\;c_{ij}=1\right\},\;t_{i}^{l}=\max\left\{t_{j},j=1,\ldots ,n_{occ}:\;c_{ij}=1\right\},$$

for *i* = 1, . . . , *n_d_*, respectively.

We used these quantities to estimate three indices already reported and applied in other studies (monthly rate, yearly rate, and periodicity; [45,46,47]) and three new indices specifically developed for this study (occurrence, resight rate, and relative span-time):

- **Occurrence** (Occ): The proportion of captures, determined by the number of times an individual was captured divided by the total number of capture occasions.

(1) $$Occ_{i}=\frac{c_{i}}{n_{occ}}.$$

This is different from the occurrence in [13] since all capture occasions are considered, not only recaptures.

- **Monthly rate** (MR): Monthly average number of sights.

(2) $$MR_{i}=\frac{\sum_{k=1}^{n_{m}}I_{M_{k}}\left(\sum_{t_{j}\in M_{k}}c_{ij}>0\right)}{n_{m}}$$

- **Yearly rate** (YR): Yearly average number of sights.

(3) $$YR_{i}=\frac{\sum_{k=1}^{n_{m}}I_{Y_{k}}\left(\sum_{t_{j}\in Y_{k}}c_{ij}>0\right)}{n_{y}}$$

- **Resight rate** (RR): The average number of recaptures over all the recapture occasions.

(4) $$RR_{i}=\frac{c_{i}-1}{t_{n_{occ}}-t_{i}^{f}}.$$

This is similar to the occurrence in [13], but the denominator considers only the recapture occasions *after* the first sight. The rationale behind this choice is that the individual may have entered the population after the first occasion.

- **Periodicity** (P): The recurrence of an individual, determined by the inverse of the average time between successive recaptures.

(5) $$P_{i}={\left(\frac{t_{i}^{l}-t_{i}^{f}}{c_{i}}\right)}^{-1}$$

- **Relative span-time** (RS): The portion of the whole observation time elapsed between the first and last captures of the individual.

(6) $$RS_{i}={\left(\frac{t_{i}^{l}-t_{i}^{f}}{t_{n_{occ}}-t_{1}}\right)}^{-1}\;$$

All variables were standardized to make different metrics comparable in the cluster analysis. The Gower dissimilarity [48] was chosen because of its broad applicability in most dissimilarity-based clustering with mixed-type variables [49]. Ward’s agglomeration method was applied as it is based on a classical sum-of-squares criterion, producing groups that minimize within-group dispersion at each binary fusion and is robust in the presence of outliers [50]. To this respect, the optimal number of clusters was selected using the heuristic *elbow rule* for the total within-cluster sum of squares. Analysis was performed in RStudio 4.0.1 [51] using factoextra package.

#### 2.3.3. Abundance Estimate

The total number of sampling (capture) occasions analyzed in this study were *n_occ_* = 99. These single-capture occasions were combined monthly, totaling *n_m_* = 20 capture occasions (4 months in 2017, 4 months in 2018, 7 months in 2019, and 5 months in 2020). The encounter history of each identified individual was then as ${\left\{{\tilde{c}}_{ij}\right\}}_{j=1}^{n_{m}}$, where ${\tilde{c}}_{ij}$ is equal to 1 if the individual *i* has been encountered at least once during the *j*-th month (0 if vice versa).

To test whether the bottlenose dolphin population at the Tiber River estuary was closed or open, the CloseTest software [52] was used, applying both Stanley and Burnham Closure Test [53] and Otis Closure Test [54]. As the population was open (Stanley and Burnham Closure Test chi-square statistic = 392.353, df = 29, *p* < 0.05; Otis Closure Test *z*-value = −13. 287, *p* < 0.05), we used the POPAN data type [55], which uses a parameterization of the Jolly–Seber model [56], to estimate its abundance. Open-population models are the most widely used to estimate the abundance of bottlenose dolphin populations [57,58,59]. These models venture the existence of a *superpopulation N*, which represents the total number of individuals that are potentially available in the study area between the first and the last sampling occasion [60,61]. The superpopulation approach [56,62], which includes inference about probabilities of entry into the sampled population and is useful for populations in which group membership is dynamic and temporally unpredictable, was used. The main assumptions are the following:

1. Captures are independent across individuals and along time.
2. Both capture probability *p_j_* (i.e., detectability) and survival probability *φ_j_* on each occasion are homogeneous among all individuals [62].
3. Each individual from the superpopulation *N* enters the study area on occasion *j* with the same probability *β_j_*; then, it stays in the study area as long as it survives, with no chance of re-entrance once it has exited.

Thus, the parameters on which each capture history depends on are as follows:

1. ${\left\{p_{j}\right\}}_{j=1}^{n_{m}}$, the capture probabilities of each occasion.
2. ${\left\{\phi_{j}\right\}}_{j=1}^{n_{m}-1}$, the survival probability between two subsequent sampling occasions; as migration/immigration phenomena are not distinguishable from natality/mortality, *φ* represents the apparent survival probability.
3. ${\left\{\beta_{j}\right\}}_{j=1}^{n_{m}-1}$, the probability that an individual from the superpopulation will join the population between two subsequent sampling occasions.

The parameters may be constant or may vary over time, also depending on specific variables (e.g., effort). Parameters’ estimation was carried out maximizing the likelihood of a multinomial distribution (as the observed data are represented by the capture histories), and the following models were obtained:

- Survival probability between two subsequent sampling occasions:
  − **Constant:** It does not vary across groups or over time: *φ*.
  − **Occasion:** It does not vary across groups but can vary over time: *φ_t_*, *t* = 1, …, *n_m_*.
  − **Group:** It does not vary over time but can vary by group: *φ_g_*, *g* = 1, …, *G*.
- Detectability at each sampling occasion:
  − **Constant:** It does not vary across groups or over time: *p*.
  − **Log-effort:** It does not vary over time but depends linearly on the logarithm of the effort: *p_l e f f_*, where *l e f f* = *log*(*effort*) and *effort* ∈ ${\mathbb{N}}^{+}$
  − **Occasion:** It does not vary across groups but can vary over time: *p_t_*, *t* = 1, …, *n_m_*.
- Entry probability between two subsequent sampling occasions:
  − **Constant:** It does not vary across groups or over time: *β*.
  − **Occasion:** It does not vary across groups but can vary over time: *β*_t_, t = 1, …, *n_m_*.
  − **Group:** It does not vary over time but can vary by group: *β_g_*, *g* = 1, …, *G*.

To avoid biases [63] and produce accurate estimates of the parameters, the following assumptions were strictly considered [56]:

- Marks are not lost over time, and individuals are identified with no error [63].
- Sampling is simultaneous, and each individual is released just after.
- Captures do not affect the detectability and survival probability of the captured individuals after release.

Considering the rigorous data collection procedures and selection process used for photo-identification described in Section 2.2 and Section 2.3.1, we deemed these assumptions appropriate. However, for a gregarious species like bottlenose dolphin, overdispersion is observed most of the time because the survival of an individual living in a group is not independent from the fate of the others [64]. The Cormack–Jolly–Seber (CJS) statistics provides a measure, which we refer to as $\hat{c}$, that indicates the amount of underdispersion ($\hat{c}$ *<* 1) or overdispersion ($\hat{c}$ *>* 1) displayed by the data. When $\hat{c}$ = 1, the model’s choice using the simple AIC (Akaike’s information criterion) may be flawed. A more accurate metric for model comparison, as suggested in [65], is represented by the QAICc (quasi-likelihood Akaike’s information criterion), defined as

(7) $$\mathrm{QAICc}=-\frac{2\;\log\left(L\right)}{\hat{c}}+2k+\frac{2k\left(k+1\right)}{n-k-1}$$

where *L* stands for the likelihood, *n* is the sample numerosity, and *k* is the number of parameters in the model. QAICc proves to be robust if $\hat{c}$ ≤ 3. Larger values of $\hat{c}$ better support models with few parameters [66]. The best model is the one that minimizes (7), but models with a QAICc difference (ΔQAICc) of less than 2 provide the same inference. Finally, the superpopulation *N*, which considers well-marked (WM), fairly marked (FM), and unmarked (UM) dolphins (see Section 2.3), was calculated adjusting its estimates with the mark rate *θ* [67]:

(8) $$\hat{\theta }=\frac{n_{WM}}{n_{WM}+n_{FM}+n_{UM}}$$

where *n_WM_*, *n_FM_*, and *n_UM_* are the number of WM, FM, and UM sampled individuals, respectively. We can subsequently use $\hat{\theta }$ to correct the estimate of *N* as

(9) $$\hat{N_{tot}}=\frac{\hat{N}}{\hat{\theta }}.\;$$

The uncertainty was evaluated as suggested in [67], and confidence intervals were derived assuming log-Normal distribution as proposed in [68].

The goodness-of-fit (GOF) test of the standard CJS model, which combines the hypothesis of equal capture and survival probabilities among the individuals (homogeneity) and of closeness of the population, was used. If the test is statistically significant (i.e., *p* < 0.05), the model does not adequately fit the data. All analyses were performed in RStudio 4.0.1 [51] using R2ucare and RMark packages.

## 3. Results

A total of 137 surveys were conducted between august 2017 and november 2020, covering 4967 km in the study area (Figure 1), and a total number of 105 bottlenose dolphin groups were encountered. The group size was variable (mean: 15; range: 1–65), with an average identification rate of 13.78 (±1.13 SE) per sighting. Effective photo-identification work was performed on 99 out of the 105 groups; in three encounters, animals were very elusive and we decided to not chase them; during the other three sightings, we were not able to collect useful pictures (*Q* < 4). A total number of 104,781 images were selected for photo-identification out of the 263,703 pictures that were collected over the study period, identifying 347 unique individuals. The maximum number of re-sightings was 30 for a single individual, with 226 animals encountered only once or twice.

### 3.1. Site Fidelity

**Figure 2:**
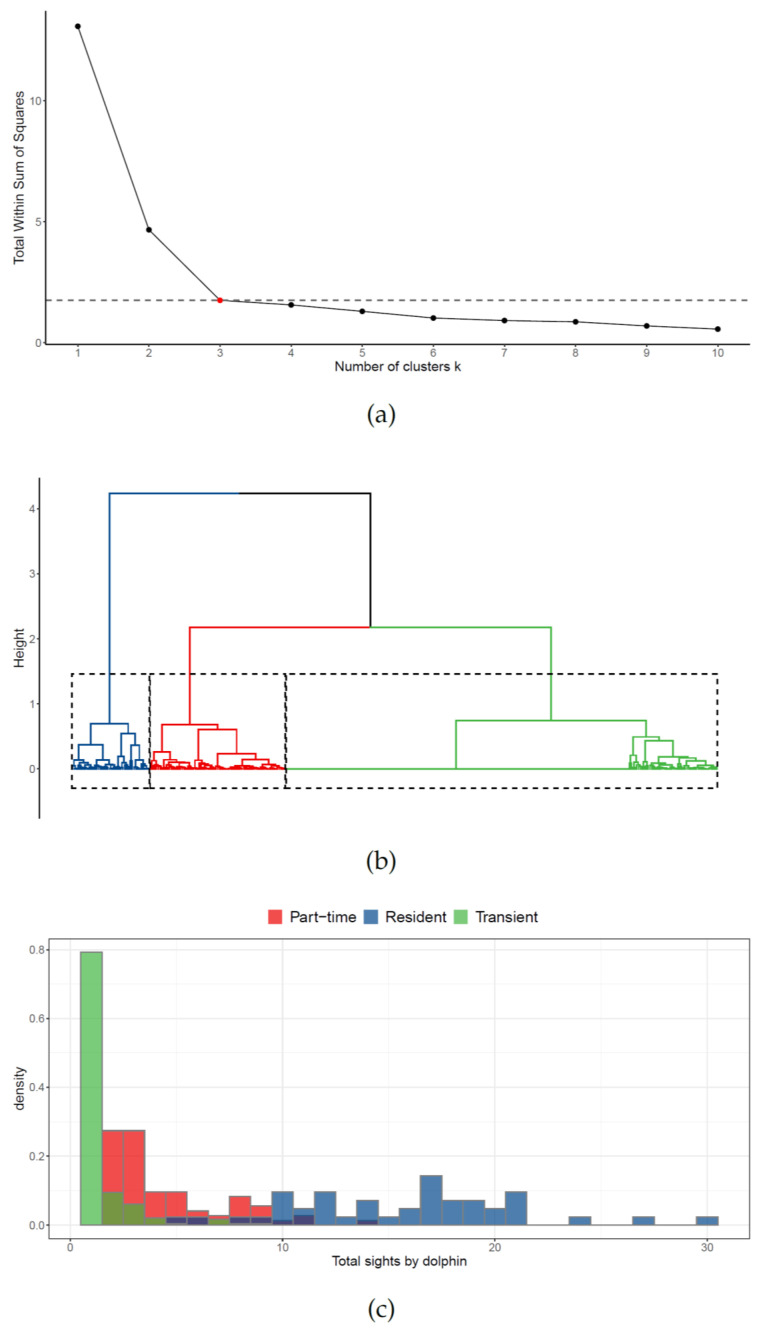
Results of the cluster analysis: (**a**) optimal number of clusters; (**b**) cluster dendrogram; (**c**) distributions of total individual identifications by cluster.

The hierarchical cluster analysis, used to describe the presence of possible dolphin groups according to their site-fidelity patterns in the surveyed area, identified an optimal number of *G* = 3 clusters (Figure 2a). The resulting dendrogram (Figure 2b) clearly highlighted three well-separated groups, which have been labeled resident (in blue), part-time (in red), and transient (in green). Each cluster was identified and characterized using the corresponding centroids of the site-fidelity metrics (see Table 1). The number of encounters per individual in the three different clusters is reported in Figure 2c.

The first cluster, accounting for 42 dolphins, was characterized by the largest values of all site-fidelity metrics, except for periodicity. Thus, it was categorized as resident. Individuals belonging to this cluster were more likely to be seen very frequently (in absolute number of times) in the study area, but not necessarily on close sampling occasions. In particular, they were encountered at least five times, in three different months, over three distinct years. The number of encounters ranged from 5 to 30, with more than 50% of the individuals (24/42 dolphins) identified at least 15 times. None of the Resident dolphins were sighted all surveyed months, but 17 (40.4%) were regularly encountered over the 4-year study period. Overall, the maximum number of resident dolphins encountered per sighting was recorded in September (about 9.5 dolphins per sighting).

The second cluster, accounting for 73 dolphins, was identified as the group of part-time dolphins. This cluster ranked second in all the site-fidelity metrics, except for periodicity. This means that individuals in this group were likely to be seen at distant occasions in time; they were encountered at least on two occasions in a month, in at least two different years. The number of encounters ranged from 2 to 14, with more than 50% of the individuals (47/73 dolphins) sighted fewer than five times. The number of months ranged from 1 up to 5, with about 50% of the individuals (37/73) encountered at least in three different months. Only 11 individuals (15.1%) were identified in three different years, and none of them in all surveyed years. Overall, the maximum number of part-time individuals encountered per sighting was recorded in October (about 5 dolphins per sighting).

The third cluster was the most numerous, accounting for 232 dolphins, and showed the largest average periodicity but the lowest values for all the other site-fidelity metrics (see Table 1). A large value of the periodicity means that animals were mostly encountered on very close dates, if sighted more than one time. Individuals belonging to this cluster were identified as transient dolphins, as only 48 (20.1%) were encountered on more than one occasion, 36 (15.5%) in more than 1 month, and none in more than 1 year. The overall maximum number of transient individuals encountered per sighting was recorded in October (about 4.5 dolphins per sighting).

Although the number of females was almost equally distributed among the three clusters (resident = 18; part-time = 16; transient = 15; total = 49), the relative contribution was higher for resident (43%) than for the other clusters (part-time: 22%; transient: 6%). No new females were identified in 2020. We were only able to positively recognize one male, meaning that all other individuals in each cluster were classified as unknown.

### 3.2. Abundance Estimates

The goodness-of-fit test was significant (*χ*^2^ = 153.68; *p* < 0.05, df = 58), and this could bias estimates; hence, estimates were adjusted with the variation inflation factor ($\hat{c}$ = 2.65). The four models providing the best fit in terms of QAICc score are reported in Table 2, together with the number of free parameters *k* and abundance estimates.

The model with varying detectability by occasion, varying survival, and entry probability by group (Model 1, *φ_g_*, *p_t_*, *β_g_*) attained the lowest QAICc value (i.e., the best score). This model includes 18 parameters more than Model 2 (*φ_g_*, *p_l e f f_*, *β_g_*), in which the detectability varies only as an effect of the effort. Negligible differences in terms of point and interval estimates between these two models emerged, and the ∆QAICc was only slightly larger than the threshold suggested in [64]. Hence, we deemed Model 2 to be a better and more parsimonious choice for interpretable modeling of the evident heterogeneity in capture, survival, and entry probabilities. The other two reported models will not be discussed in detail because of their larger ∆QAICc. Nevertheless, these provide very similar inference in terms of the final abundance estimates, both at the overall and group levels. Yearly mark rates are reported in Table 3.

Given their evident inter-year variability, mark rates were used to adjust the yearly abundance estimates derived by the overall POPAN model (see Section 2.3.3). The superpopulation (${\hat{N}}_{tot}$) of bottlenose dolphins spanning the entire study period (4 years) estimated by Model 2 and adjusted for the mark rate ($\hat{\theta }$ = 0.58) is 529 individuals (*CI*_0.95_ = (456; 614)), with 80 residents (*CI*_0.95_ = (67; 95)), 114 part-time (*CI*_0.95_ = (100; 132)), and 335 transients (*CI*_0.95_ = (282; 397)).

**Figure 3:**
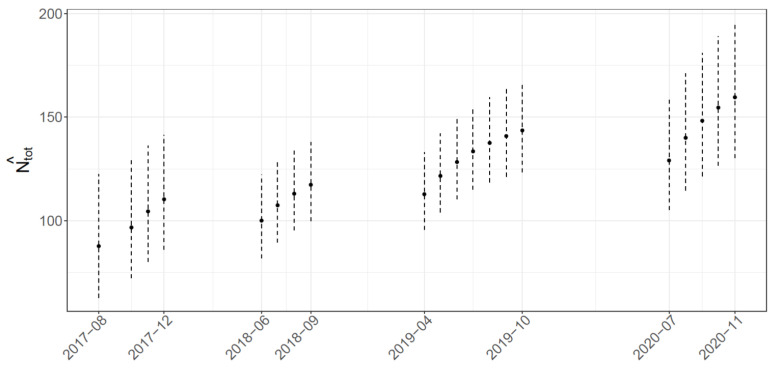
Estimated abundance by each sampling occasion.

Abundance estimates on each sampling occasion (Figure 3) show an increasing trend within and between the surveyed years, although not statistically significant. In particular, estimates range from 88 (*CI*_0.95_ = (63; 122)) to 110 (*CI*_0.95_ = (86; 141)) in 2017, from 100 (*CI*_0.95_ = (82; 122)) to 117 (*CI*_0.95_ = (100; 138)) in 2018, from 113 (*CI*_0.95_ = (96; 133)) to 144 (*CI*_0.95_ = (123; 167)) in 2019, and from 129 (*CI*_0.95_ = (105; 158)) to 159 (*CI*_0.95_ = (130; 195)) in 2020.

**Figure 4:**
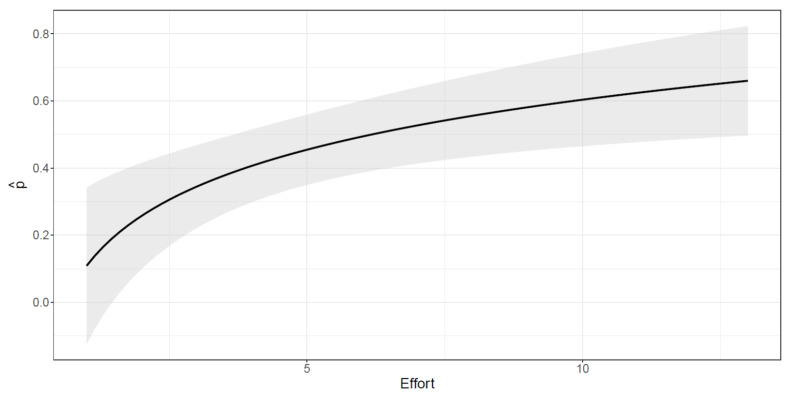
Estimated capture probability as a function of the effort (total monthly surveyed days).

The capture probability varies with the effort (Figure 4); however, the increase in the detectability induced by a unit increment in the effort is less relevant as the effort increases. The asymptote is reached after 10–12 sampling days, meaning that differences in the capture probability after this threshold are negligible.

The apparent survival rate is 0.703 (*CI*_0.95_ = (0.604; 0.786)) for transient, 0.987 (*CI*_0.95_ = (0.976; 0.933)) for part-time, and 0.975 (*CI*_0.95_ = (0.962; 0.984)) for resident. The probability of entering the superpopulation for the part-time dolphins is 3.5% and for the transient ones is 5.3%. It is obviously 0 for the resident.

## 4. Discussion

This study reports the most updated, comprehensive analysis of the site-fidelity patterns and size of the bottlenose dolphin population at the Tiber River estuary, in central Mediterranean. Such information has never been reported before, as only data on distribution in the region were available in the literature [26]. Understanding the degree of residency and providing robust estimates of bottlenose dolphin population abundance are necessary for assessing baseline conditions, evaluating population trends, and developing management strategies. Having the potential of being immediately used by wildlife managers, the results of this study may offer a significant contribution to the comprehension of the status of the species in the Roman waters and add basic knowledge for conservation purposes.

### 4.1. Site Fidelity

Bottlenose dolphin populations show different patterns of residency worldwide, with some displaying large movements and low site fidelity [10,58,69] and others short-range patterns and strong site fidelity [70,71,72]. Low levels of site fidelity are often typical of individuals inhabiting oceanic zones or areas characterized by low productivity and unpredictable prey availability [73], while higher levels are usually displayed by bottlenose dolphins in protected coastal areas, where prey biomass is more recurrent and foreseeable in distribution [19,72,74]. Our results suggest that the population using the area of the Tiber River estuary is made up of three groups of individuals, classified here as resident, part-time, and transient, who show different degrees of site fidelity.

The resident group (42 dolphins) has a very localized distribution and a lower number of individuals compared to the transient and part-time groups, with almost 50% of them identified as females (the gender of the other 50% is unknown). Females that show strong site fidelity and limited movements have been reported as typical of bottlenose dolphin populations living close to estuaries [75,76], as in our case. Females with restricted home range, low levels of dispersion, and high levels of site fidelity have been described for other marine mammal species as well (e.g., gray seal, *Halichoerus grypus* [77]; sperm whale, *Physeter macrocephalus* [78]; Indo-Pacific bottlenose dolphins, *Tursiops aduncus* [71]). However, data limitation does not allow us to infer with confidence whether resident dolphins remain at the Roman shores all year round or are present only part of the year or whether the study area represents only part of their home range. The group of part-time dolphins accounts for more individuals (73 dolphins) compared to the resident one, with 20% recognized as females (the gender of the remaining 80% is unknown). This cluster is mainly composed of dolphins that temporary left the study area, showing larger-scale movements into and out of the region over the study period, apparently using it occasionally. The most abundant group is the transient one (232 dolphins), principally composed of individuals encountered only once. These individuals are likely to use the area as a crossing site, spending there just one or few close dates. Recognizable females represent 6% of group members (the gender of the others is unknown).

The inclusion of each individual in the resident, part-time, or transient group may change with additional data [79]; however, animals that are currently clustered as resident are unlikely to change category, while the group assignment may be more variable for transient and part-time, considering the higher flexibility of these categorizations. Apparently, ranging patterns can change over time in response to environmental changes, such as prey distribution [80], and human activities, such as dolphin-watching [81] and mariculture [82]. Continuing to monitor residency of Capitoline dolphins could provide an indirect measure of habitat quality in a rapidly changing coastal environment.

### 4.2. Abundance Estimates

This study provides the first empirical estimates of abundance of bottlenose dolphins at the Tiber River estuary. The overall mark rate ($\hat{\theta }$) of this population is equal to 0.585, similar to those given by other studies [73,83,84,85,86], with peaks higher than 0.70, as in [87,88]. However, the annual mark rate decreases through the investigated years, with the lowest value in 2020 ($\hat{\theta }$ = 0.53). We suggest that this trend may be related to the increasing number of juveniles, less marked than adults, that entered the population over time and the influence of low-marked transient individuals captured in 2020.

Under the parsimony principle in the POPAN model selection, our results indicate that bottlenose dolphins are abundant in the study area. The estimated abundance of the superpopulation is 529 individuals (*CI*_0.95_ = (456; 614)), similar to that of the North Adriatic Sea [59]. However, the heterogeneous survey effort in the study area (see Figure 1), due to different constraints (e.g., distance from the berth place of the research vessel, weather forcing, distribution of the fishing gears), is likely to have influenced the abundance estimate. In this respect, under-coverage of the whole range of the population may imply that individuals may have been traveling into or out of the area, inducing some bias in the final estimates. As the survey effort was not uniformly distributed across the study area, this population estimate should be considered as a minimum [10].

The estimates calculated for each occasion vary from August 2017 to November 2020 and seem to reveal a growth in the total number of individuals within and between years (see Figure 3). This increasing trend cannot be explained solely as an effect of the augmented effort across the years, since it is already considered in the model in order to adjust the estimates. On the other hand, it is possibly related to the higher probability of capturing part-time or transient individuals over time. It is reasonable to assume that these individuals travel longer distances than resident, possibly showing large-scale movements of hundreds of kilometers, as observed in other Mediterranean locations (e.g., 265 km in the Gulf of Corinth [89] and 467 km in the Pelagos Sanctuary [90]). In addition, the fluctuations in abundance observed in our study may be related to different habitat use as well, which is mainly influenced by reproduction, prey availability, and disturbance [83,91,92,93]. A combination of these factors may explain the increasing number of dolphins we measured at the end of each survey season (September–October), when fishery with trawling vessels is prohibited for almost 30 days to preserve marine resources and allow the recovery of commercial target species. Disturbance of and pressure on demersal prey by fishing are reported to strongly affect the abundance of bottlenose dolphins [59]. This seems coherent with the resident individuals in our study area, which show higher presence in September, probably as a consequence of the interruption in fishing activities. However, in other Mediterranean sites [94], the abundance of bottlenose dolphins seems to decrease with the absence of trawling vessels, possibly because of the lower site fidelity shown by the local population [57]. Reduced disturbance by maritime traffic [95,96] and underwater noise [97] caused by the pandemic emergency in 2020 may have further contributed to an increase in the number of individuals in this coastal area.

The presence of resident individuals and the estimated abundance suggest that the area surrounding the Tiber River estuary, despite its proximity to one of the major urban centers in the world, represents an important, suitable habitat for bottlenose dolphins. In addition, the significant number of females with calves showing strong site fidelity could also suggest habitat suitability for breeding and calving, as reported for other cetaceans [98,99]) and bottlenose dolphins as well [20]. River mouths are known as important feeding areas for opportunistic species, such as bottlenose dolphin, since nutrient transport influences primary production and the whole trophic web [100]. The Tiber River is one of the most polluted rivers in Italy [101], with high heavy-metal concentrations, organophosphate pesticide pollution, and solid waste reported around the mouths [102]. These facts indicate that this coastal bottlenose dolphin population may be highly susceptible to variations in local ecological/environmental conditions and highlight the importance of correctly managing and controlling the potential impacts by human activities [26]. As apex predators and K-selected species with very low reproductive rates [103,104], bottlenose dolphins are sensitive to threats such as pollutants, habitat degradation, reduced prey availability, and disturbance from vessel traffic and anthropogenic noise, which may lead to demographic effects on the population. The investigation of the population parameters and inferences on how risks might affect them emphasize the necessity for the realization of management actions ensuring that dolphins habitats may improve and not deteriorate [105].

## 5. Conclusions

The abundance estimates and the apparent survival rates assessed here (over 97% for both resident and part-time individuals, as expected for slowly reproducing and long-living mammals, such as bottlenose dolphin [40,106]) may be effectively used as a tool to support concrete management measures. Given the high number of individuals in the study area, we suggest that (1) collaborative effort at all levels (resources, data, and expertise) be developed to address the multiplicity of problems confronting the species [106] and (2) conservation plans not be focused only close to the Tiber River mouths but be extended to cover a broader scale of area. For example, outspreading the coverage of the already existing MPA (regulating both fishing and boating activities) and defining the Central Tyrrhenian Sea as an Important Marine Mammal Area (IMMA) in the Mediterranean (https://www.marinemammalhabitat.org/imma-eatlas/) (accessed on 25 February 2021) would be beneficial for the conservation of the ecology and genetic diversity of bottlenose dolphins in this region. In this potential scenario, information reported by the present work, focused both at population and individual levels, could support the evaluation of these proposals.

## Figures and Tables

**Table 1:** Cluster numerosity and centroids of the site-fidelity metrics: occurrence (Occ), monthly rate (MR), yearly rate (YR), re-sighting rate (RR), periodicity (P), and relative span-time (RS).

Cluster	No. of Dolphins	Occ	MR	YR	RR	P	RS
Resident	42	0.167	0.416	0.851	0.173	0.017	0.738
Part-time	73	0.048	0.160	0.541	0.052	0.008	0.382
Transient	232	0.016	0.062	0.250	0.013	0.019	0.008

**Table 2:** Best models in terms of quasi-likelihood Akaike’s information criterion (QAICc) with the corresponding number of parameters *k*. Point estimates (and 95% confidence interval) of the WM (${\hat{N}}_{WM}$ ) and overall (${\hat{N}}_{tot}$ ) population by group.

Model	K	QAICc	ΔQAICc	Cluster	${\hat{N}}_{WM}$	${\hat{N}}_{tot}$
1: *Φ_g_*, *p_t_*, *β_g_*	27	648.02	0	Resident	45 (39, 52)	77 (65, 91)
				Part-time	65 (59, 72)	111 (97, 128)
				Transient	202 (173, 235)	354 (288, 312)
				Total	312 (276, 353)	533 (458, 621)
2: *Φ_g_*, *p_l e f f_*, *β_g_*	9	651.88	3.86	Resident	47 (40, 54)	80 (67, 95)
				Part-time	67 (60, 75)	114 (100, 132)
				Transient	196 (169, 227)	335 (282, 397)
				Total	310 (275, 349)	529 (456, 614)
3: *Φ_g_*, *p_t_*, *β_t_*	43	683.86	35.84	Resident	38 (31, 46)	65 (53, 80)
				Part-time	58 (51, 66)	99 (85, 116)
				Transient	196 (164, 236)	336 (274, 412)
				Total	292 (252, 339)	500 (421, 593)
4: *Φ_g_*, *p_l e f f_*, *β*	7	696.56	48.55	Resident	47 (42, 52)	80 (69, 92)
				Part-time	67 (62, 73)	115 (102, 129)
				Transient	187 (163, 216)	320 (271, 379)
				Total	301 (271, 335)	514 (448, 591)

**Table 3:** Overall and yearly mark rate ($\hat{\theta }$ ).

	Overall	2017	2018	2019	2020
$\hat{\theta }$	0.585	0.786	0.752	0.64	0.534

## Data Availability

The data presented in this study are available to any qualified researcher on request from the corresponding author.
